# Dataset on seston and zooplankton fatty-acid compositions, zooplankton and phytoplankton biomass, and environmental conditions of coastal and offshore waters of the northern Baltic Sea

**DOI:** 10.1016/j.dib.2022.108158

**Published:** 2022-04-10

**Authors:** Tharindu Bandara, Sonia Brugel, Agneta Andersson, Danny Chun Pong Lau

**Affiliations:** a Department of Ecology and Environmental Science, Umeå University, 90187 Umeå, Sweden; b Department of Animal Science, Faculty of Animal Science and Export Agriculture, Uva Wellassa University, Passara Road, 90000 Badulla, Sri Lanka; c Umeå Marine Sciences Centre, Umeå University, 90571 Hörnefors, Sweden; d Department of Aquatic Sciences and Assessment, Swedish University of Agricultural Sciences,75007 Uppsala, Sweden

**Keywords:** *Eurytemora affinis*, Cladocerans, Dha, Epa, Food web quality, DHA, EPA

## Abstract

We analyzed the taxonomic and fatty-acid (FA) compositions of phytoplankton and zooplankton, and the environmental conditions at three coastal and offshore stations of the northern Baltic Sea. Plankton samples for FA analyses were collected under the framework of sampling campaigns of the Swedish National Marine Monitoring program in September 2017. Monitoring data of phytoplankton and zooplankton biomass, and environmental variables at each station were extracted from the Swedish Meteorological and Hydrological Institute database (https://sharkweb.smhi.se/). Monthly phytoplankton biomass at each station in July-September 2017 was aggregated by class (i.e., chyrsophytes, cryptophytes, dinoflagellates, diatoms, euglenophytes, cyanobacteria, etc.). Zooplankton biomass in September 2017 was aggregated by major taxa (i.e., *Acartia* sp. [Calanoida], *Eurytemora affinis* [Calanoida], Cladocera, *Limnocalanus macrurus* and other copepods (i.e. excluding *Eurytemora* and *Acartia*)). Environmental variables monthly monitored in January-October 2017 included salinity, concentrations of dissolved organic carbon, humic substances, total nitrogen and total phosphorus. These variables were measured from 0 to 10 m depth below water surface, and the depth-integrated averages were used for data analyses. Seston and zooplankton (*Eurytemora affinis, Acartia* sp. and Cladocera) FA compositions were analyzed using gas chromatography and mass spectroscopy (GC–MS). Our dataset could provide new insights into how taxonomic composition and biochemical quality of the planktonic food chains change with the environmental conditions in subarctic marine ecosystems.

## Specifications Table

**Table:** 

Subject	Environmental Science
Specific subject area	Marine Ecology
Type of data	Tables Figure
How the data were acquired	Seston and zooplankton samples for fatty-acid (FA) analyses were collected from multiple stations in the northern Baltic Sea. Sample FA were analyzed using a gas chromatography interfaced with a mass-spectrometry (instruments: 7890A GC, 185 Agilent Technologies, CA, United States; Pegasus® High Throughput TOF–MS, MI, United States) following the methods in [2,3]. Phytoplankton and zooplankton biomass and the environmental parameters are secondary data and extracted from the Swedish Meteorological and Hydrological Institute database (https://sharkweb.smhi.se/), that contains long-term data collected by the Swedish National Marine Monitoring program following the HELCOM guidelines [4].
Data format	Raw Analyzed
Description of data collection	At each station, phytoplankton and zooplankton for biomass and FA analyses were collected from depth-integrated samples following the HELCOM guidelines [4]. Phytoplankton was sampled by a hose (0–10 m from water surface), and zooplankton by vertical net hauls with a WP2 plankton net of 90 µm mesh size. Environmental parameters were measured by the Swedish National Marine Monitoring program following the HELCOM guidelines [4] and collected from https://sharkweb.smhi.se/ database.
Data source location	Country: Sweden Latitude and longitude for collected samples: Zooplankton and seston for FA analyses were collected from the following 3 stations in the northern Baltic Sea. • Station: A5 (65° 10′ N, 23° 14′ E, depth 90 m) • Station: B3 (63° 30′ N, 19° 49′ E, depth 25 m) • Station: C14 (62° 06′ N, 18° 33′ E, depth 85 m) Primary data sources for phytoplankton and zooplankton biomass, and the environmental parameters: the Swedish Meteorological and Hydrological Institute database (https://sharkweb.smhi.se/), for the 5 stations below. • Station: A5 (65° 10′ N, 23° 14′ E) • Station: A13 (64° 43′ N, 22° 04′ E) • Station: B3 (63° 30′ N, 19° 49′ E) • Station: C14 (62° 06′ N, 18° 33′ E) • Station: C3 (62° 39′ N, 18° 57′ E)
Data accessibility	Public repository Repository name: Mendeley data Data identification number: doi: 10.17632/rvt3zf6gnj.1 Direct URL to data: https://data.mendeley.com/datasets/rvt3zf6gnj/1
Related research article	[1] T. Bandara, S. Brugel, A. Andersson, D.C.P. Lau, Seawater browning alters community composition and reduces nutritional quality of plankton in a sub-Arctic marine ecosystem, Can. J. Fish. Aquat. Sci. (2022). https://doi.org/10.1139/cjfas-2021–0118

## Value of the Data

• The data on the ratios between essential polyunsaturated FA and non-essential monounsaturated FA in zooplankton (*Eurytemora affinis* and cladocerans) reflected the spatial changes in taxonomic and FA compositions of phytoplankton caused by the environmental gradients in the northern Baltic Sea. These FA ratios in zooplankton are potential indicators of marine environmental changes.
• FA provide a unique account of the biochemical quality and health of zooplankton, which are important prey of fish. Thus, the data are useful for researchers and environmental managers whose concerns are on the impacts of environmental stressors on the quality and health of food webs and fish production in marine ecosystems.
• Data can be re-used for meta-analysis, FA-based mixing models and other studies on environmental effects on plankton FA composition. Similarly, data can be used in hindcast models that determine aquatic food web quality and studies on trophic transfer efficiency in aquatic ecosystems.

## 1 Data Description

The raw data of FA compositions of seston and zooplankton are available at https://data.mendeley.com/datasets/rvt3zf6gnj/1. Table 1 describes the monthly phytoplankton biomass (µg C m^−3^) collected from the sampling stations in July-September 2017. Chrysophytes, cryptophytes, dinophytes, and euglenophytes are rich in docosahexaenoic acid (DHA), while diatoms are rich in eicosapentaenoic acid (EPA) according to [5,6]. Table 2 describes the monthly environmental variables measured at the sampling stations in July-September 2017. These data were averaged by depth (0–10 m from the water surface). Table 3 indicates the total wet zooplankton biomass (g m^−^^2^) and relative zooplankton biomass at the sampling stations in September 2017.

**Table 1:** Monthly phytoplankton biomass at the sampling stations in the northern Baltic Sea in July-September 2017. At A5 and C14 stations phytoplankton were not regularly monitored and therefore data were not available in July and August 2017.

		Phytoplankton biomass (µg C m^-3^)						
Month	Station	Chrysophytes	Cryptophytes	Dinophytes	Euglenophytes	other	Diatoms	Cyanobacteria
July	A5							
August	A5							
September	A5	0	814	0	0	5760	2527	879
July	B3	29	1000	1718	0	7077	7035	12,949
August	B3	1895	1837	784	7	14,912	908	6415
September	B3	140	2247	6831	807	5760	17,743	23,507
July	C14							
August	C14							
September	C14	731	6399	23,098	1072	5651	1827	9969
July	A13	3131	1699	0	0	31,783	5699	535
August	A13	3225	2295	0	0	18,358	7809	574
September	A13	0	6490	0	147	38,618	4740	2371
July	C3	9939	672	15,372	701	36,308	5762	13,417
August	C3	611	7247	5808	0	33,365	641	108,802
September	C3	324	5472	17,861	0	8733	6533	8273

**Table 2:** Monthly environmental variables at the sampling stations in the northern Baltic Sea in July-September 2017. Environmental variables were averaged by depth (0–10 m from the water surface). Values are mean ± SD, with *n* = 3. Salinity, Total N, Total P and DOC data at A13 and C3 stations are not indicated but can be accessible via https://sharkweb.smhi.se/hamta-data/. DOC and humic substances data were not available in August 2017 at A5 and C14 stations.

		Environmental variables				
Date	Station	Salinity (psu)	Total N (μM)	Total P (μM)	DOC (μM)	Humic substances (μg l^−1^)
July	A5	2.85	18.25±0.18	0.22±0.005	386.00	15.60
August	A5	2.54±0.012	15.78±0.24	0.17		
September	A5	2.66±0.04	17.89±0.28	0.17±0.005	370.00±4.24	16.20
July	B3	3.73±0.65	16.30±0.20	0.35	398.50±0.70	15.00
August	B3	4.21±0.25	17.33±1.2	0.43±0.05	385.00±4.24	12.60±0.14
September	B3	3.69±0.015	16.75±0.40	0.36±0.025	363.00	15.05
July	C14	5.18±0.004	16.78±0.12	0.33±0.015	338.00±4.24	8.00
August	C14	4.90±0.003	19.37±0.28	0.44±0.0005		
September	C14	5.05±0.0006	15.85±0.44	0.30±0.005	331.50±4.94	8.05±0.07
July	A13					15.05
August	A13					15.1
September	A13					14.9
July	C3					8.2
August	C3					8.4
September	C3					8.65

**Table 3:** Zooplankton biomass (wet weight) at the sampling stations in the northern Baltic Sea in September 2017.

	% Zooplankton biomass				
Station	Cladocera	Copepod (others)	*Eurytemora Affinis*	*Limnocalanus Macrurus*	Total zooplankton biomass (g m^−2^)
A5	19.00	2.46	15.70	62.84	21.81
A13	13.04	4.39	20.23	62.34	17.35
B3	43.70	14.50	36.41	5.40	9.20
C3	14.31	25.46	23.86	36.37	45.64
C14	16.97	19.47	33.48	30.08	37.69

## 2 Experimental Design, Materials and Methods

### 2.1 Sample collection for fatty-acid (FA) analyses

**Fig 1:**
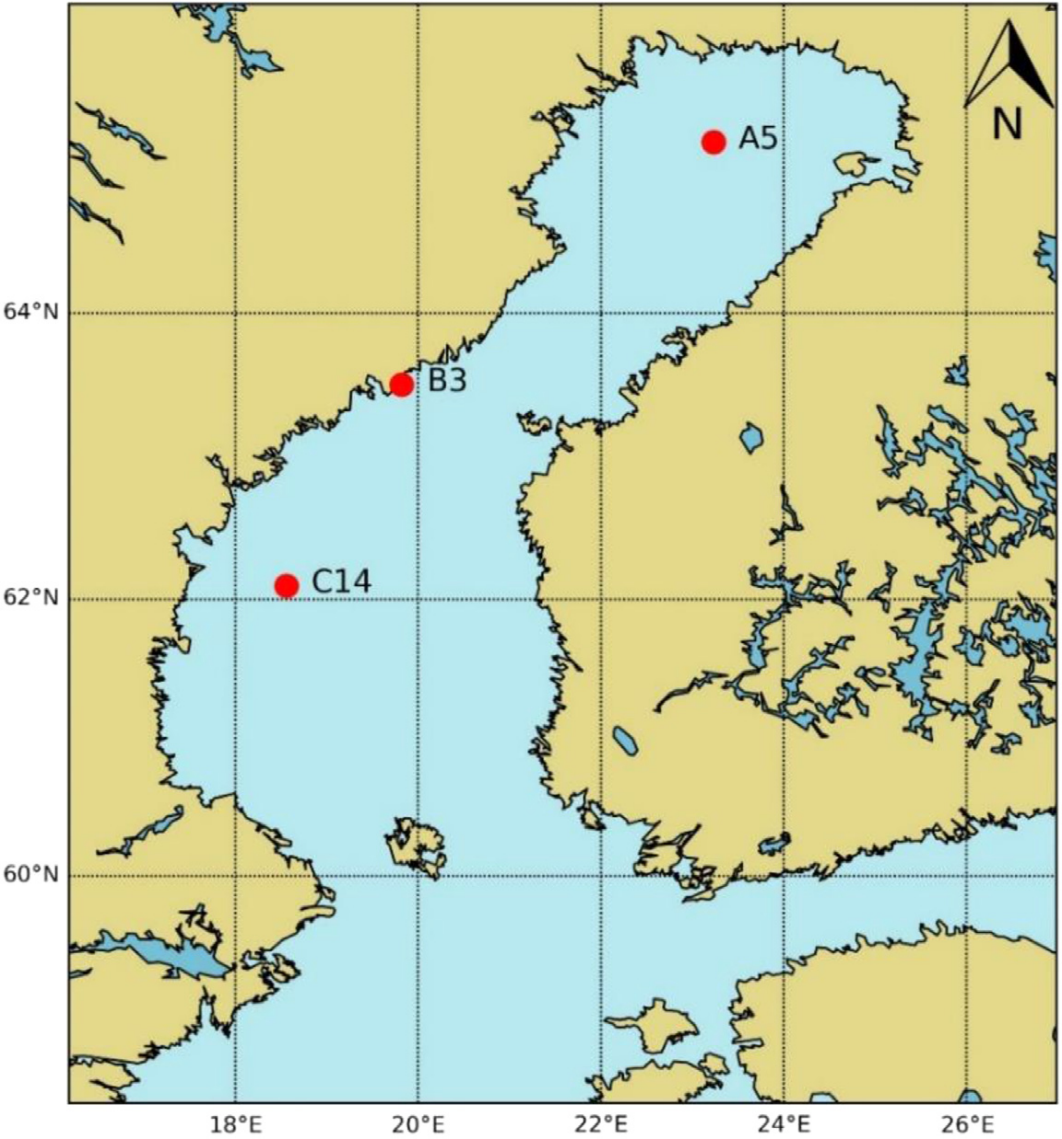
Sampling locations in the northern Baltic Sea (modified from [1]).

In August-September 2017, three stations (A5, B3, and C14) in the northern Baltic Sea were sampled to understand the impact of increased terrestrial organic matter on the nutritional quality in planktonic food chains. Station A5 (65° 10′ N, 23° 14′ E, depth 90 m) is located in the Bothnian Bay, while station B3 (63° 30’ N, 19° 49’ E, depth 25 m) and C14 (62° 06′ N, 18° 33′ E, depth 85 m) are in the Bothnian Sea (Fig 1).

We collected seston and zooplankton samples for FA analysis following the HELCOM guidelines during the sampling campaigns of the Swedish National Marine Monitoring program [4]. Seston was collected from water sampled with an integrated hose from 0 to 10 m below water surface. 300 ml of the collected water was filtered onto pre-combusted (5 h at 450°C) GF/F filters with a diameter of 25 mm (Whatman,GE healthcare, USA). The filters were then frozen at −20°C and later freeze-dried before FA extraction.

Zooplankton were collected by using four vertical net hauls of the whole water column with a WP2 plankton net of 90 µm mesh size. Sampling was conducted in August-September 2017 (summer) which is the major growth season of zooplankton. Collected zooplankton were starved for at least 16 h in filtered (0.2 µm) natural seawater at 4°C in dark to empty their gut content. Subsequently, mesozooplankton was sorted according to taxa. We particularly selected three zooplankton taxa, i.e., *Eurytemora affinis* (Poppe, 1880) (Copepoda: Temoridae) *Acartia* sp. (Copepoda: Acartiidae) and cladocerans (mostly *Bosmina coregoni maritima* (Müller P.E., 1867) (Phyllopoda: Bosminidae)), because of their wide distribution in the Baltic Sea and their differences in essential FA requirement [7]. The sorted taxa were frozen at −20°C and then freeze-dried before FA extraction.

### 2.2 FA analyses

The FA composition of seston and zooplankton was analyzed following a modified method as described in [2,3] and the detailed procedure for the analysis can be found in [1]. Briefly, the FA of 5 mg of homogenized freeze-dried sample were extracted using 3:2 (v:v) hexane-isopropanol solution. The deuterium-labeled D_29_-pentadecanoic acid (120 ng µl-1; C/D/N isotopes, Essex, UK) was used as the internal standard. Methylation of the samples was done by using 1:17:83 (v:v:v) trimethylsilyldiazomethane:isopropanol:dichloromethane.

We quantified the concentrations of FA methyl esters in the samples by using a gas chromatography-mass spectrometry (7890A GC, Agilent Technologies, CA, United States; Pegasus® High Throughput TOF–MS, MI, United States) installed with a DB-5 capillary column (length 30 m, internal diameter 250 μm, film thickness 0.25 μm; Agilent Technologies). Splitless injection of 1 μl was used for each sample. Detailed inlet and oven temperature programs are reported in [2]. Individual FA were identified by using the Supelco 37 Component FAME Mix (Sigma-Aldrich Sweden AB, Stockholm, Sweden) and the Bacterial Acid Methyl Ester BAME Mix (Sigma-Aldrich Sweden AB). Concentrations of individual FA in the samples are reported as proportions (%) of total FA.

### 2.3 Environmental variables and biomass of phytoplankton and zooplankton

The environmental conditions as well as phytoplankton and zooplankton community compositions and biomass were monthly measured at the sampling sites following the HELCOM guidelines from January to October 2017 [4]. These measurements were conducted during the sampling campaigns of the Swedish National Marine Monitoring program. Measured environmental variables included: salinity, dissolved organic carbon (DOC), humic substances, total phosphorus (Total P), and total nitrogen (Total N). In situ salinity was measured by a Sea-Bird SBE 19 plus CTD. Sea water samples for DOC concentration measurements were filtered through Supor membrane syringe filters (0.2 µm pore size, non-pyrogenic, Acrodisc®, Pall), acidified (18 mM HCl, final concentration) and analyzed using a high temeprature combustion Shimadzu TOC-5000 analyser. The concentration of humic substances was measured from unfiltered water samples using a Perkin Elmer LS 50 spectrofluorometer at 350/450 nm excitation/emission wavelengths, calibrated with a serial dilution of quinine sulfate solution [8]. Water samples for Total N and Total P were analyzed using the Seal QuAAtro39 auto-analyzer after an oxidation step using peroxodisulfate [9].

Measurements were averaged over depth, i.e. from 0 m to 10 m below water surface. DOC and humic substances concentrations were used to indicate the browning level of seawater, as DOC is a measure of colored and non-colored dissolved organic carbon, whereas humic substances are a measure of the colored fraction of dissolved and particulate organic matter.

Depth-integrated water samples for phytoplankton analyses were collected from 0 to 10 m depth below the water surface using a vertical hose, and preserved in acidic Lugol solution (2%). Samples were settled for 24–48 h and counted using the inverted microscope method [10]. Phytoplankton biomass was calculated from the geometric shape of cells following [11] and cell carbon content was calculated according to [12].

Zooplankton for community composition and biomass measurements were sampled from the whole water column with vertical net hauls from 5 m above the bottom with a WP2 plankton net of 90 µm mesh size. The samples were preserved in 4% formaldehyde solution and sub-sampled with Stempel-pipette before identification using a stereomicroscope. The wet weight for the different taxonomic groups and developmental stages were calculated according to [13].

All of these data are available at the Swedish National Marine Monitoring program database at https://sharkweb.smhi.se/.

Zooplankton accumulate fatty acids in their active growth season, i.e. summer. Therefore, we used phytoplankton data of our study stations from July-September 2017. At each station, the monthly biomass data (µg C m^-3^) of phytoplankton were aggregated at the class level. The phytoplankton classes included chrysophytes, cryptophytes, dinoflagellates, diatoms, euglenophytes, and cyanobacteria. Similarly, the biomass data of individual zooplankton species were collected in September 2017 and aggregated into the following taxa: *Acartia* sp., *Eurytemora affinis*, cladocerans, *Limnocalanus macrurus*, and other copepods.

## Ethics Statements

There is no ethical issue for this study as no animals or patients were involved in data acquisition.

## CRediT authorship contribution statement

**Tharindu Bandara:** Data curation, Visualization, Investigation, Writing – original draft. **Sonia Brugel:** Data curation, Methodology, Investigation, Writing – review & editing. **Agneta Andersson:** Conceptualization, Methodology, Writing – review & editing, Supervision. **Danny Chun Pong Lau:** Conceptualization, Methodology, Investigation, Writing – review & editing, Supervision.

## Declaration of Competing Interest

The authors declare that they have no known competing financial interests or personal relationships that could have appeared to influence the work reported in this paper.

## Data Availability

Dataset on seston and zooplankton fatty-acid composition: coastal and offshore waters of the northern Baltic Sea (Original data) (Mendeley Data).
